# Vascular Signaling in Allogenic Solid Organ Transplantation – The Role of Endothelial Cells

**DOI:** 10.3389/fphys.2020.00443

**Published:** 2020-05-08

**Authors:** Laura Kummer, Marcin Zaradzki, Vijith Vijayan, Rawa Arif, Markus A. Weigand, Stephan Immenschuh, Andreas H. Wagner, Jan Larmann

**Affiliations:** ^1^Department of Anesthesiology, University Hospital Heidelberg, Heidelberg, Germany; ^2^Institute of Cardiac Surgery, University Hospital Heidelberg, Heidelberg, Germany; ^3^Institute for Transfusion Medicine, Hannover Medical School, Hanover, Germany; ^4^Institute of Physiology and Pathophysiology, Heidelberg University, Heidelberg, Germany

**Keywords:** endothelial activation, donor-specific antibodies, transplant vasculopathy, vascular signaling, HLA I and II

## Abstract

Graft rejection remains the major obstacle after vascularized solid organ transplantation. Endothelial cells, which form the interface between the transplanted graft and the host’s immunity, are the first target for host immune cells. During acute cellular rejection endothelial cells are directly attacked by HLA I and II-recognizing NK cells, macrophages, and T cells, and activation of the complement system leads to endothelial cell lysis. The established forms of immunosuppressive therapy provide effective treatment options, but the treatment of chronic rejection of solid organs remains challenging. Chronic rejection is mainly based on production of donor-specific antibodies that induce endothelial cell activation—a condition which phenotypically resembles chronic inflammation. Activated endothelial cells produce chemokines, and expression of adhesion molecules increases. Due to this pro-inflammatory microenvironment, leukocytes are recruited and transmigrate from the bloodstream across the endothelial monolayer into the vessel wall. This mononuclear infiltrate is a hallmark of transplant vasculopathy. Furthermore, expression profiles of different cytokines serve as clinical markers for the patient’s outcome. Besides their effects on immune cells, activated endothelial cells support the migration and proliferation of vascular smooth muscle cells. In turn, muscle cell recruitment leads to neointima formation followed by reduction in organ perfusion and eventually results in tissue injury. Activation of endothelial cells involves antibody ligation to the surface of endothelial cells. Subsequently, intracellular signaling pathways are initiated. These signaling cascades may serve as targets to prevent or treat adverse effects in antibody-activated endothelial cells. Preventive or therapeutic strategies for chronic rejection can be investigated in sophisticated mouse models of transplant vasculopathy, mimicking interactions between immune cells and endothelium.

## Introduction

Endothelial cells (ECs) are semiprofessional antigen-presenting cells; furthermore they express all major sets of antigens that can be recognized by immune cells. Therefore, they constitute a preferential target in vascularized grafts for the host immune system to discriminate between self and non-self (Piotti et al., 2014). Various transplantation-dependent factors lead to EC activation, and upon reperfusion ECs themselves trigger T cell co-stimulation and specific immune cell activation. It has been shown *in vitro* that the co-stimulation properties of ECs are influenced by their vascular origin, the presented antigen, and the maturity of the T cell (Rothermel et al., 2004). So far, rejection after allogeneic solid organ transplantation remains the major limiting factor for graft survival. Allograft rejection can be categorized as hyperacute, acute, or chronic, depending on the time of onset after the transplant procedure. In addition, it can be classified on the basis of the principal mechanism, such as cell-mediated or antibody-mediated rejection.

### Preformed Antibodies Against ECs Elicit Hyperacute Rejection

In vascularized grafts, hyperacute rejection is seen within minutes after organ reperfusion. The underlying mechanism is the presence of preformed anti-donor specific antibodies in the recipient prior to transplantation (Moreau et al., 2013). Common reasons for these preformed antibodies are previous blood transfusions, transplantations, and in women, a history of one or more pregnancies. The preformed anti-donor specific antibodies are directed against ECs and other vascular cells. Deposition of antibodies on the EC surface is sufficient to activate the complement system, both distinct mechanisms result in formation of an interstitial neutrophilic infiltrate, intravascular platelet adhesion, and aggregation. One observation, specific for hyperacute rejection after lung transplantation, is diffuse alveolar damage promoted by donor-specific IgG antibodies that induce T cell-mediated lymphocytotoxicity (Frost et al., 1996). In addition to its effects on immune cells and platelets, the activated complement system initiates an enzymatic cascade that forms the membrane attack complex (MAC), resulting in pores in the plasma membrane of ECs and subsequent cell lysis (Wehner et al., 2007). Nowadays hyperacute organ rejection has become rare because the detection of anti-donor specific antibodies is a routine procedure performed before any organ transplantation (Moreau et al., 2013).

### T Cell- and B Cell-Dependent Pathways Contribute to Acute Rejection

Whereas hyperacute rejection occurs within the first few minutes after organ reperfusion, acute rejection refers to graft rejection days or months after transplantation (Mengel et al., 2012). While features of adaptive immunity are used to describe and characterize acute rejection, the innate immune system also plays a crucial role in acute transplant rejection. Importantly, its effects are in part independent of adaptive immunity. For example, in mice lacking an adaptive immune system but developing normal NK and myeloid cell compartments, pro-inflammatory cytokines, such as interleukin-1β (IL-1β) and interleukin-6 (IL-6), are significantly upregulated after heterotopic heart transplantation (He et al., 2003). Besides several immunological factors there are various non-immunological factors, e.g., ischemia–reperfusion (I/R) injury or infections during transplantation, that are harmful to graft ECs (Chong and Alegre, 2012; Krezdorn et al., 2017). Similar to hyperacute rejection, acute rejection can arise in a T cell-mediated fashion, the so-called acute cellular rejection or in a B cell-dependent mechanism termed antibody-mediated rejection. The two mechanisms can occur independently of each other, but the immunological pathways of acute cellular rejection and antibody-mediated rejection overlap (Moreau et al., 2013). In acute cellular rejection, there are two known antigen-dependent T cell-activating pathways. In the direct pathway, T cells of the host immune system recognize intact foreign HLA: antigen complexes presented on the surface of donor-derived antigen presenting cells (APCs) in the host lymphoid organs. In contrast, in the indirect pathway, recipient T cells recognize fragments of donor HLA peptides bound to HLA molecules on recipient APCs (Ochando et al., 2006). Both pathways contribute to B cell activation which plays a crucial role in developing antibody-mediated rejection. Antibody-mediated rejection is driven by generation of antibodies directed against HLA I and HLA II molecules or other immunogenic targets on the surface of graft ECs. In early antibody-mediated rejection, *de novo* synthesized donor-specific antibodies against HLA I and HLA II molecules are equally common. During late antibody-mediated rejection, however, donor-specific antibodies are mainly directed against HLA II molecules. This finding is interpreted as an indicator for two distinct pathways in the development of antibody-mediated rejection (Walsh et al., 2011). Persistent occurrence of antibodies against the graft endothelium results in chronic antibody-mediated rejection.

The past few years have seen improvements in immunosuppressive therapies and concepts to tackle acute rejection. As a result, acute rejection is now seen in less than 15% of patients that lack preformed anti-donor specific antibodies. With fewer episodes of acute rejection and improved short-term graft survival, chronic rejection has become increasingly relevant (Najarian et al., 1985; Gonzalez-Molina et al., 2014).

### Chronic Rejection Arises From Persistent Inflammation of the Endothelium

Chronic allograft rejection develops over a period of months to years and is described as transplant vasculopathy (TV), characterized by neointima formation. With further progression, the luminal diameter decreases and the internal elastic lamina is destroyed. Intima thickening, as a hallmark of TV, is manifested by proliferation of myofibroblasts and accumulation of extracellular matrix, both seen on histopathological examination. TV is found as bronchiolitis obliterans syndrome (BOS) in lung transplantation, as cardiac allograft vasculopathy after cardiac transplantation, and as renal transplant arteriosclerosis following kidney transplantation (Pedagogos et al., 1997; Pilmore et al., 2000). One risk factor for the development of chronic rejection is the occurrence of donor-specific antibodies. In a prospective, single-center cohort study, 47% of the patients were serum positive for antibodies against graft ECs after lung transplantation (Tikkanen et al., 2016). This agreed with an earlier study’s finding of a negative correlation between the appearance of anti-donor specific antibodies and graft survival (Mao et al., 2007). Antibodies against the major histocompatibility complex (MHC) I can elicit chronic allograft rejection in mice lacking functional T and B cells (Uehara et al., 2007). Even in the absence of an intact complement system, one of the generally accepted criteria for antibody-mediated rejection, a mononuclear infiltrate is formed by NK cells and macrophages (Hirohashi et al., 2010).

**FIGURE 1:**
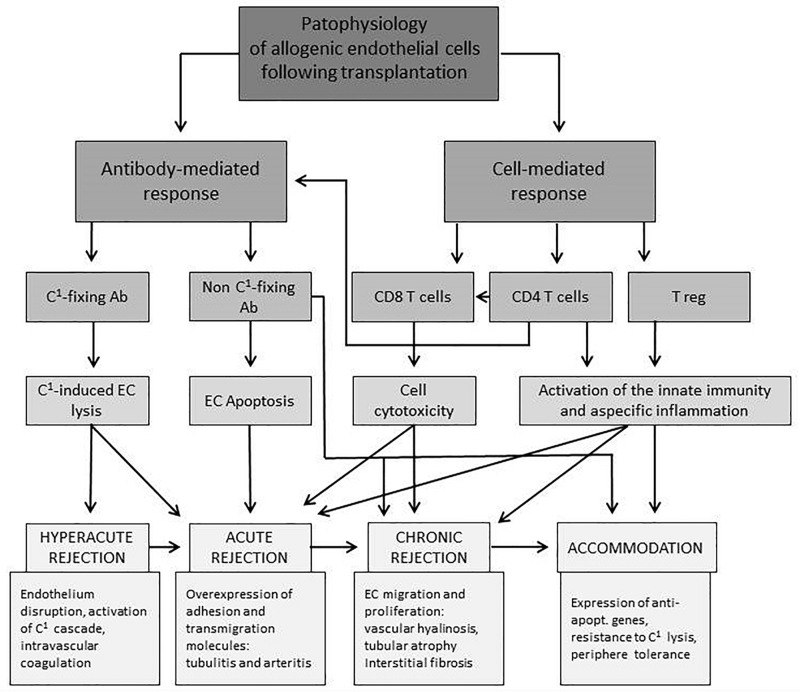
Overview of interactions of endothelial and immune cells leading to different forms of rejection after solid organ transplantation. EC, endothelial cell; C^1^, complement factor; Ab, antibody; expr., expression; apopt., apoptotic (Piotti et al., 2014).

Figure 1 provides an overview of the interplay of different cellular compartments of the innate and adaptive immune system as well as soluble factors such as antibodies and complement factors. All of the pathways, starting with allorecognition of the graft and leading to rejection, interfere with others. The time of occurrence and concentration of each factor determine the phenotype of rejection.

In acute inflammation, ECs undergo transcriptional and translational changes and are converted into an activated state. Activated ECs are phenotypically characterized by increased permeability and cytokine release, enhanced adhesiveness for leukocytes, and pro-thrombic features (Pober and Cotran, 1990). These reactions serve to effectively eliminate invading pathogens and destroy potentially harmful agents. However, when the immune system fails to resolve inflammation a chronic inflammatory state will persist, involving subsequent destruction of primarily unaffected tissue (Ryan and Majno, 1977). Altogether, ECs in a transplanted solid organ can be activated during the surgical procedure of transplantation, either by presenting antigens bound to their HLA molecules or by antigens expressed by themselves.

The major questions we address in this review include the following:

• What are the main target structures on the vascular endothelium of the transplanted organ that can be recognized by immunological and non-immunological factors?
• How will the endothelial phenotype be affected during activation?
• What role plays the immune system during activation of ECs and in organ rejection?
• How is the vascular structure altered due to organ rejection?
• What kind of research models do we have to address further questions, and what are the advantages and limitations of the different models?

## Immunological Endothelial Activation Factors

A major goal after solid organ transplantation remains prevention of an ongoing inflammatory process in the vessel wall, which is the pathological correlate of chronic rejection. Therefore, a reduced donor-specific immune response in a mature immune system is desirable. Graft-infiltrating, innate immune cells comprise a major pro-inflammatory stimulus driving TV and putting graft function at risk.

### Endothelial Interactions of Anti-HLA Antibodies

It is established that antibodies against molecules of the major histocompatibility complex (MHC), which is termed human leukocyte antigen (HLA) in humans, play a critical role in transplant rejection after solid organ transplantation via fixation and activation of complement, which in turn causes cytotoxicity in the graft endothelium (Patel and Terasaki, 1969). In addition to these so-called complement-dependent effects, more recent evidence suggests that ligation of anti-HLA antibodies can also cause complement-independent effects in the graft endothelium via induction of intracellular signaling cascades (Thomas et al., 2015). In particular, binding of anti-HLA class I (HLA I) antibodies has been shown to cause phenotypical alterations of the endothelium, including pro-inflammatory activation via up-regulation of inducible pro-inflammatory adhesion molecules and cytokines such as intercellular cell adhesion molecule-1 (ICAM-1), vascular adhesion molecule-1 (VCAM-1), and monocyte chemoattractant protein-1 (MCP-1) (Naemi et al., 2013; Zilian et al., 2015), as well as increased adhesion of inflammatory leukocytes via Fcγ receptor (FcγR)-dependent mechanisms (Hirohashi et al., 2012; Valenzuela et al., 2013b). Moreover, binding of anti-HLA I antibodies has been associated with proliferation of ECs (Jindra et al., 2008; Thomas et al., 2015). The complement-independent effects of anti-HLA antibodies in ECs are mediated via activation of a variety of signaling cascades including, but not limited to, mitogen-activated protein (MAP) kinase pathway, the extracellular-regulated kinase (ERK) pathway, and the nuclear factor (NF)-kappa B and fibroblast growth factor (FGF) pathway (Thomas et al., 2015). Another important intracellular signal transducer in ECs is mechanistic target of rapamycin (mTOR). HLA I crosslinking on ECs triggers mTOR/Rictor/Sin1 association, which results in formation of mTORC2 complex (Jin et al., 2014). Rearrangement of the cytoskeleton and cell migration is mediated through activation of mTORC2 and the downstream-located Rho GTPases. Furthermore, anti-HLA I antibodies mediate mTORC1 formation by inducing the mTOR-Raptor complex, resulting in increased EC proliferation (Sarbassov et al., 2004). Binding of anti-HLA I antibodies induces phosphorylation of Akt at Ser473 and ERK at Thr202/Tyr204, inducing expression of the anti-apoptotic genes Bcl-2 and Bcl-xL (Jin et al., 2004). Another way for anti-HLA I antibodies to induce EC proliferation is via the generation of inositol phosphate, which serves as a messenger of Akt signaling (Bian et al., 1997). *In vitro* treatment of ECs with the mTOR inhibitors sirolimus and everolimus reduces monocyte adhesion by repressing mTORC1- and mTORC2-dependent pathways. Accordingly, administering mTOR inhibitors in a mouse model of fully mismatched cardiac transplantation results in reduced mononuclear infiltration (Salehi et al., 2018). Furthermore, anti-HLA I antibodies induce tyrosine phosphorylation of members of the Src family, regulating complex signal transduction pathways (Jin et al., 2002). Activated Src is required for phosphorylation of cortactin, an actin-binding molecule, which is part of the adhesion molecule ICAM-1 cluster. Phosphorylated cortactin stabilizes ICAM-1 clusters and induces cytoskeletal remodeling with improved leukocyte transmigration capacity (Yang et al., 2006). In contrast with the regulatory events mediated by anti-HLA I antibodies in the endothelium, the effects of anti-HLA II antibodies are less well established. Le Bas-Bernadet and colleagues demonstrated that the monoclonal HLA-DR antibody L243 caused differential effects in human vascular ECs and B cells, such as activation of the protein kinase C and protein kinase B/Akt signaling cascades (Le Bas-Bernardet et al., 2004). A more recent report demonstrated that HLA II antibody-dependent interaction with human ECs induced a complex TH17 cell-dependent immunological mechanism that might mediate humoral kidney transplant rejection. Specifically, endothelial ligation of a monoclonal anti-HLA II antibody and native allospecific anti-HLA II antibodies from patient sera activated this pathway via up-regulation of interleukin (IL)-6 in a co-culture model of a human EC line and primary peripheral blood monocytes (Lion et al., 2016). Independently, Zhang and colleagues have demonstrated that endothelial HLA II ligation caused proliferation and migration of ECs via the induction of a complex network of signaling cascades including Src, focal adhesion kinase, phosphatidyl-inositol-3 kinase (PI3K), and ERK (Jin et al., 2018). Finally, the monoclonal anti-HLA II antibody L243 and native anti-HLA II antibodies from allosera have recently been shown to cause complement-independent non-apoptotic cytotoxicity in human ECs via a lysosomal membrane-mediated cell death pathway (Aljabri et al., 2019).

### Endothelial Interactions of Non-HLA Antibodies

Numerous experimental and clinical studies have demonstrated that antibodies directed against endothelial non-HLA antigens are also critically involved in acute and chronic AMR after transplantation of various solid organs (Opelz, 2005). However, compared with anti-HLA antibodies, much less is known on the generation and functional significance of non-HLA antibodies in transplant rejection.

Two major groups of non-HLA antibodies are known. The first group is directed against polymorphic alloantigens, whereas the second group interacts with a variety of autoantigens of the endothelium (Zhang and Reed, 2016). A prototypical alloantigen targeted by antibodies of the first group is the endothelial MHC I chain-related gene A (MICA) (Zou and Stastny, 2009). The clinical importance of antibodies against MICA has been demonstrated in a study on kidney transplant patients (Zou et al., 2007). Non-HLA antibodies of the second group are directed against numerous endothelial autoantigens, including several cell surface or intracellular proteins (Dragun et al., 2016). Importantly, non-HLA antibodies directed against autoantigens appear to be of major clinical significance, because their presence in the circulation is associated with adverse clinical outcome, as recently reported by independent groups for renal transplantation (Cardinal et al., 2017; Delville et al., 2019; Lefaucheur et al., 2019). For example, an autoantigen targeted by non-HLA antibodies is the G protein-coupled receptor anti-angiotensin type I receptor (AT1R), which is critical for mediating the effects of angiotensin II in blood vessels (Dragun et al., 2016). The clinical significance of AT1R antibodies for rejection has been demonstrated in kidney transplantation patients (Dragun et al., 2005). Other examples of autoantigens targeted by non-HLA antibodies include the endothelial receptor endothelin type A receptor (ET1AR), perlecan, and endoglin (Dragun et al., 2016). Interestingly, a large number of other non-HLA candidate proteins that may serve as endothelial autoantigens associated with transplant rejection have been identified by array approaches (Li et al., 2009; Sigdel et al., 2012). The mechanisms by which non-HLA antibodies mediate transplant rejection are currently under intense investigation. Similar to what has been explained for complement-independent signaling of HLA antibodies in the endothelium, non-HLA antibodies may mediate their detrimental effects in transplantation via the induction of endothelial signal transduction (Zhang and Reed, 2009). An important issue for future studies will be to improve our understanding of the interrelationship of HLA alloantibodies and non-HLA autoantibodies in the pathogenesis of humoral rejection.

**FIGURE 2:**
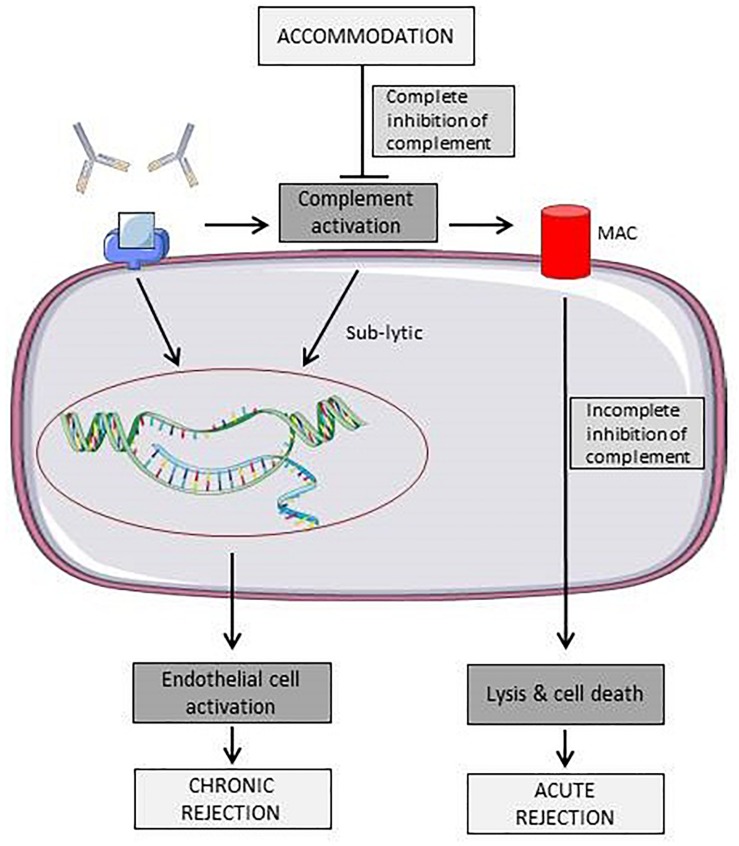
Schematic representation of known effects of antibodies towards surface antigens on endothelial cells. Binding of antibodies can either leads to acute rejection due to immune cell or complement mediated lysis or to a state of chronic rejection due to endothelial cell activation (Colvin and Smith, 2005). Elements of Figures 2, 3 and 4 were taken and adjusted from Servier Medical Art at http://smart.servier.com, licensed under a Creative Commons Attribution 3.0 Unported License.

Figure 2 shows different effects of antibodies towards ECs. Due to complement activation, antibodies may induce acute rejection of the graft by directly damaging the endothelium or, if the antibody titer reaches a sub-lytic level, EC expression profile is altered, leading to a more chronic rejection phenotype.

## Non-Immunological Endothelial Activation Factors

Non-immunological factors activating the endothelium of vascular allografts are still not fully understood but have been best investigated in cardiac, lung and renal transplantation. Activation of ECs is a multifactorial process that is regularly initiated long before the donor’s brain death. Factors associated with critical illness, pain, infections, and treatment contribute to EC activation. When brain death is diagnosed, the therapeutic goals are revised with the aim of protecting organs from further adverse events. Still, factors such as I/R injury and systemic inflammatory reaction caused by the artificial surface of the cardiopulmonary bypass (during heart transplantation) contribute to ongoing endothelial injury.

### Brain Death of Organ Donors Is the First Inducer of Endothelial Dysfunction During the Process of Transplantation

Organ donors are predominantly diagnosed with brain death due to cerebral damage following intracranial bleeding or trauma (McKeown et al., 2012). During this process, before organ retrieval is initiated, the donor organism undergoes profound systemic changes. Consequently, approximately 25% of potential organ donors are excluded from explantation due to hemodynamic instability (Szabo, 2004; Girlanda, 2016). Furthermore, the process of organ retrieval—as a multi-visceral operation—also causes systemic inflammation, altering vascular structures, which requires intensive hemodynamic management to minimize the risk of organ hypoperfusion, arrhythmia or cardiac arrest.

Acute cerebral damage is immediately followed by a rapid increase of intracranial pressure and is compensated by a catecholamine storm, resulting in arterial hypertension and bradycardia (Smith, 2004). The acute catecholamine-mediated compensatory mechanisms are followed by a loss of sympathetic activity and consecutive peripheral vasodilatation with the risk of hypoperfusion of possible allografts. Changes of plasma catecholamines during the late phase after brain death result in endothelial dysfunction (Szabo et al., 2002). Szabo et al. established a canine model of induced brain death (inflation of a subdural balloon) to assess coronary blood flow and the influence of the endothelium on vasodilatation. Besides changes in blood flow, the authors observed severe endothelial dysfunction by impaired vasodilation caused by application of endothelium-dependent acetylcholine (Szabo et al., 2002). The same group demonstrated in a large animal model, that coronary blood flow increases approximately threefold but drops significantly below baseline levels as soon as the acute phase is over. They hypothesized that nitric oxide supply improves endothelial function, because after infusion of L-arginine, the substrate for nitric oxide supply, the decrease in coronary blood flow was less pronounced. They concluded that enhancement of endogenous nitric oxide synthesis due to L-arginine treatment is beneficial for endothelial function and thus for myocardial performance after brain death (Szabo et al., 2006).

Takada et al. (1998) showed in a rat model, that experimentally induced explosive brain death is followed by an up-regulation of immunoregulatory and cell adhesion molecules (CAMs) compared to animals with non-explosive brain death). They postulated a preconditioning effect on allografts leading to adverse donor–host reactions after transplantation. Segel et al. found an abundance of CAMs and increased cytokine expression in animal models of brain death and searched for an association with endothelial dysfunction. Relative expression of ICAM-1, VCAM-1, IL-1, and IL-6 mRNAs was significantly elevated in brain-dead animals, while the hemodynamics remained uncompromised (Segel et al., 2002). The authors concluded that an increase in IL-1 might mediate the overexpression of the adhesion molecules and IL-6 mRNAs. Similar effects have been proven for humans by Mehra et al., who divided recipients into groups that received cardiac allografts from donors with either explosive or non-explosive brain death (EBD vs. non-EBD). EBD was defined as acutely increased intracranial pressure (Mehra et al., 2004). No significant differences were found in posttransplant survival and distribution of immunological and non-immunological variables between recipients of organs from EBD donors and recipients of organs from non-EBD donors. Interestingly, allografts from EBD donors demonstrated advanced intimal thickening and a higher cardiac event rate, by contrast with grafts from non-EBD donors. Consequently, hearts from donors with EBD had lower organ survival than those from non-EBD donors. These findings were attributed to a release of cytokines following leukocyte activation in vascular beds of all peripheral organs including the heart (Mehra et al., 2004).

Koo et al. (1999) found lower E-selectin, DR locus of HLA (HLA-DR), ICAM-1, and VCAM-1 expression in biopsies from human living-related kidney donors than from cadaveric donors, which may be associated with beneficial graft survival). Similar findings were also observed in cadaveric and living-donor livers before transplantation (Jassem et al., 2003). These results are further supported by Anyanwu et al. (2002) investigating domino hearts (living-related heart transplantation from recipients who require heart-lung transplantation). Domino hearts also tend to develop less allograft vasculopathy than cadaveric grafts.

*Ex vivo* lung perfusion models have widely been used to assess endothelial activation during transplantation in lungs. Park et al. showed in a xenotransplant model, that nitric oxide donor treatment reduced platelet adhesion and vascular resistance of the lung (Park et al., 2015). Von Willebrand factor (vWF) secretion from ECs was reduced; complement activation and thrombin generation were inhibited. Another treatment strategy to prevent EC activation was investigated by Kim et al., who showed that aurintricarboxylic acid (ATA), a platelet inhibitor, significantly inhibited tumor necrosis factor alpha (TNF-α)- or lipopolysaccharide-induced endothelial E-selectin expression. As a result of inhibited E-selectin expression, adhesiveness of monocyte to ECs was impeded (Kim et al., 2008). Thrombin-induced vWF secretion and complement activation were reduced, although *in vitro* findings revealed that ATA induced endothelial tissue factor expression and platelet activation (Kim et al., 2008).

Altogether, ECs of transplanted organs are already affected during brain death and before the process of transplantation is initiated. This leads to EC activation and facilitates increased leukocyte–endothelial interactions. Thus, treatment of organ donors prior to explantation focuses on prevention of vascular allograft injury and needs to be developed further.

### I/R Injury Contributes to Further Endothelial Activation After Transplantation

Another non-immunological factor that causes endothelial dysfunction is I/R injury. Reperfusion injury develops hours or days after the initial phase of blood flow suppression or disruption during organ explantation and occurs either as cold or warm ischemia. Despite restoration of flow, further tissue and microcirculation injury occurs during reperfusion. The associated damage even exceeds the injury during the initial ischemic phase. Within the damaged tissue, apoptosis, autophagy, and necrosis are induced concurrently to start repair and regeneration processes. Predomination of regeneration processes leads to organ survival, while prevailing damaging processes result in organ failure (Nordling et al., 2018).

A common feature of graft I/R injury is increased vascular permeability caused by endothelial dysfunction and microvascular damage. The most important factor is the adhesion of neutrophils to the activated endothelium. Neutrophil adhesion to ECs is mediated by interactions between CAMs on the surface of neutrophils and ECs, e.g., P-selectin, E-selectin, and ICAM-1. The abundance of these factors depends strongly on the local tissue conditions after explantation, e.g., time of ischemia (Tsukimori et al., 2008). P-selectin expression occurs acutely following I/R injury due to its storage in preformed intracellular Weibel–Palade bodies, whereas expression of other CAMs is delayed depending on their translation process.

Neutrophil–EC adherence not only provides physical interactions but also results in altered intracellular signaling in both cell populations (Saragih et al., 2014). For instance, adhesion of neutrophils to ECs induces intracellular Ca^2+^ increases, F-actin stress fiber formation, myosin light chain kinase activation, and isometric tension generation in ECs (Wang and Doerschuk, 2000). In addition to these structural changes in the endothelium, neutrophil adherence to activated ECs induces reactive oxygen species (ROS) production only in ECs, not in neutrophils. Due to increased ROS production, neutrophil–EC interactions lead to typical necrosis (Francis and Baynosa, 2017). This appears to mediate cytoskeletal remodeling, which may stimulate subsequent inflammatory responses.

Ischemic injury and the subsequent interaction between immune cells and ECs cannot fully explain the damage observed during I/R injury. Several non-immunological conditions play a pivotal role. This includes pro-coagulatory and pro-thrombotic changes on the surface of the endothelium, resulting in vascular occlusion (Nordling et al., 2015).

Recent studies have shown that a healthy EC layer is the most important factor in maintaining proper control over inflammation and hemostasis, as described above. Alphonsus and Rodseth showed that the endothelial glycocalyx (eGC) modulates vascular homeostasis through its physical barrier properties (Alphonsus and Rodseth, 2014). Mounting evidence suggests that I/R injury causes the degradation of eGC, associated with postischemic oxidative stress and increased leukocyte and platelet adhesion. ROS may account for damage to the eGC as well (Kolarova et al., 2014). In patients suffering from sepsis, an ablated layer of eGC is negatively correlated with leukocyte–endothelial interactions, thrombogenicity, and vascular permeability. These effects could be reversed when the eGC was restored. Degradation of eGC reinforces plasminogen activator inhibitor-1 release and ICAM-1 expression, with the consequence of intensified attachment of monocytes to ECs. Furthermore, reduced eGC is associated with increased endothelial nitric oxide synthase (eNOS) activity, which is associated with impaired vascular homeostasis. These findings illustrate that physical factors also make an important contribution to regulation of the vascular inflammatory responses and blood clotting function (Cao et al., 2019).

Other work has highlighted that stressed ECs release high quantities of adenosine triphosphate (ATP) and adenosine diphosphate (ADP) into the extracellular environment. These mediators act as early stimulators of inflammatory responses, which, in turn, catalyze additional platelet aggregation, resulting in microthrombus formation and further microvascular damage. ATP and ADP can also directly stimulate macrophages and neutrophils to release pro-inflammatory mediators and express leukocyte adhesion molecules (Sugimoto et al., 2009). In several rodent transplantation models, a direct linear correlation was found between cold ischemic time, I/R injury, and early allograft dysfunction. Prolonged ischemic time was associated with increased ROS production, cytokine expression, cardiomyocyte apoptosis, and caspase activity (Yun et al., 2000; Krishnadasan et al., 2004; Tanaka et al., 2005; Lemke et al., 2015).

To date, the treatment of I/R injury relies heavily on immune-modulating drugs with undesirable side effects, but recent studies suggest new therapeutic targets (Tarjus et al., 2019). After renal transplantation, many patients develop hypertension under treatment with the immunosuppressive drug tacrolimus to suppress rejection. This is a risk factor for allograft vasculopathy and lower overall patient survival, but the underlying mechanisms have not yet been completely elucidated. A decrease in production of the vasodilator nitric oxide (NO) by eNOS has been suggested to be responsible for the endothelial dysfunction and hypertension elicited by tacrolimus (Cook et al., 2009). Therefore, immunosuppressive drugs are suspected to amplify the damage to an already critically stressed and dysfunctional endothelium. One potential new therapeutic target could be the epithelial sodium channel (ENaC). Active ENaC decreases eNOS activity and therefore reduces NO release, which in turn leads to stiffer ECs. This could explain the observed prevention of renal tubular injury and renal dysfunction after kidney I/R injury in mice with endothelial αENaC deficiency. Moreover, in human ECs, pharmacological ENaC inhibition promoted eNOS coupling and activation, resulting in NO release and vasodilatation. Altogether, the authors conclude that endothelial αENaC influences vasoconstriction and vasodilatation and plays an important role in recovery from ischemic injury (Tarjus et al., 2019).

## Soluble Factors Orchestrate Interplay Between ECs and Immune Cells

### Chemokines

Besides their prominent effects on promoting signal transduction between different cell populations, chemokines are also able to induce angiogenesis and vascular remodeling (Belperio et al., 2005). Chemokines, as well as their corresponding receptors, can be expressed in a constitutive or inducible manner on leukocytes, neurons, astrocytes, epithelial cells, or ECs and on vascular smooth muscle cells (VSMCs).

In heart transplantation models of acute allograft rejection, the chemokines CCL3 and CCL5 were upregulated and the subsequent mononuclear infiltrate could be diminished by blocking the CCL3 and CCL5 receptor CCR1 (Gao et al., 2000; Horuk et al., 2001). Another chemokine that serves as immune cell recruiter into the vessel wall during rejection is ITAC. In a prospective study with patients suffering transplant coronary artery disease, elevated peripheral blood levels of ITAC were measured and could serve as a clinical marker for patients at elevated risk of developing chronic rejection (Kao et al., 2003). ITAC binds to CXCR3 receptors on immune cells, and immunohistochemical analysis showed mononuclear infiltrates of CXCR3^+^ cells within the vasculature (Kao et al., 2003). In addition to chronic rejection after cardiac transplantation, elevated levels of CXCR3 ligands were found in patients at high risk of developing chronic lung allograft dysfunction. In this setting, CXCR3 ligands serve as chemoattractants for activated T and NK cells (Shino et al., 2017). CXCR3 and its ligands are involved in a broad spectrum of inflammatory and/or vasculature-affecting diseases (e.g., atherosclerosis, hepatitis, and systemic sclerosis). Therefore, preventing CXCR3 activation might be a promising therapeutic approach to delay graft failure (Van Raemdonck et al., 2015). On the other hand, modulation of CXCR3 expression might be a therapeutic tool to orchestrate recruitment of anti-inflammatory cells with the aim of resolving the chronic inflammation state during organ rejection. Intensive research efforts are being devoted to a next-generation DNA methyltransferase inhibitor (DMTi) in breast cancer. DMTi upregulates CXCR3 ligands and recruits CD8^+^ cells into the tumor, thereby enhancing their anti-tumor immune capacity (Luo et al., 2018).

If ECs are stimulated synergistically with IL-17 and TNF-α, *in vitro* expression of the neutrophil-specific chemokines KC, MIP2α, and LIX increases and overexpression of co-stimulatory molecules such as LFA-3 or OX-40L occurs (Griffin et al., 2012). This leads to the recruitment of leukocytes with enhanced activity, reinforcing a pro-inflammatory environment. In addition, co-culturing of allogeneic CD4^+^ T cells and ECs enhances release of IL-1α by ECs. IL-1α stimulates allogeneic memory CD4^+^ T cells to produce IFN-γ and IL-17. IL-17, in turn, stimulates predominantly smooth muscle cells (SMCs) to release cytokines and to selectively recruit CCR6^+^ T cells into allograft arteries, leading to an amplification of the immune response. These cell–cell interactions lead to memory CD4^+^ T-cell proliferation and Th1/Th17 expansion and have been verified in a humanized mouse model (Rao et al., 2008).

### Damage-Associated Molecular Patterns

Damage associated molecular patterns (DAMPs) can be released by all cell types and serve as homeostatic danger signals, indicating pathological stress during transplantation or chronic rejection (Bianchi, 2007; Gallo and Gallucci, 2013). They can be recognized either by innate lymphocytes or by pattern recognition receptors (PRR) such as toll-like receptors (TLRs) (Land,2012a). It has been shown that the high-mobility group box protein-1 (HMGB1) is upregulated in a kidney I/R injury mouse model. HMGB1 can be released from apoptotic cells or actively secreted, maintaining nucleosomal structure and regulating gene transcription (Herzog et al., 2014). It induces up-regulation of adhesion molecules on ECs, which in turn intensifies leukocyte–EC interaction and finally leads to graft damage. This effect could be abolished by blocking HMGB1, and it was not seen in TLR4^–/–^ mice lacking its receptor, which suggests involvement of the TLR4 pathway in HMGB1 signal transduction (Wu et al., 2010; Chen et al., 2011). Downstream of TLR4, both mitogen-activated protein kinase 8 (MAPK8) and apoptosis signal-regulating kinase 1 (ASK1) are activated following HMGB1–TLR4 interactions, and could thus serve as new therapeutic targets to prevent apoptosis during I/R injury (Mkaddem et al., 2009). It has been demonstrated that HMGB1 can be released from necrotic ECs and cardiomyocytes in the setting of heart transplantation and activates pro-inflammatory pathways (Park et al., 2004; Rovere-Querini et al., 2004; Bell et al., 2006). Yao et al. have shown that overexpression of microRNA26a, which plays an important role in apoptosis (Zhang et al., 2010) and induces VSMC growth (Leeper et al., 2011), inhibits HMGB1 expression and decreases cardiac I/R injury (Yao et al., 2016). Further studies are needed to investigate the mechanistic pathway of microRNA26a and to make it available as a therapy.

## Transmigration of Leukocytes Across the Endothelium

During inflammation, leukocytes are actively recruited into the vessel wall to resolve the inflammatory state, so cell–cell contact between ECs and leukocytes must be established. Alongside other triggers, donor-derived vascular cells, e.g., ECs and VSMCs, produce and release ROS as well as cytokines into the extracellular environment, recruiting neutrophils and macrophages to the site of injury. In turn, recruited and activated cells themselves start to produce, inter alia, ROS, which acts as an amplification loop for immune cell stimulation (Land,2012b).

### Activation of ECs Induces Expression of Adhesion Molecules and Growth Factors

Activation of ECs leads to rapid release of vWF. Also, adhesion molecules, such as E-selectin, P-selectin, ICAM-1, and VCAM-1, are upregulated on the surface of ECs (Salom et al., 1998; Valenzuela et al., 2013a; Fenton et al., 2016). Interestingly, Fenton et al. found a decrease of E- and P-selectin expression on the endothelium in their patient cohort of heart-transplanted children compared to age- and sex-matched controls from healthy siblings. All patients had been treated with immunosuppressant and 90% with statins after heart transplantation (Fenton et al., 2016). A direct contact of dendritic cells (DCs) and ECs, provided by adhesion molecules, leads to the transfer of intact MHC:peptide complexes from activated ECs to DCs. This offers recipient DCs to present foreign MHC molecules to T cells and serves as a link between direct and indirect allorecognition (Herrera et al., 2004).

Activation of ECs is not only characterized by intensified expression of adhesion receptors but also by enhanced synthesis of numerous growth factors (PDGF, EGF, FGF, VEGF, TGF-β, etc.) and synthesis of endothelin I (ET-1) as well as expression of the corresponding receptors (Bian and Reed, 2001; Chen et al., 2001; Rossini et al., 2005).

The presence of higher numbers of FGF receptors (FGFR) on the surface of ECs facilitates increased binding capacity of FGF, which activates the MAPK/ERK pathway and results in enhanced EC proliferation (Jin et al., 2007). ET-1 is one of the most potent vasoconstrictors in humans, and its antagonists are used to treat pulmonary arterial hypertension, but it also exerts pro-inflammatory effects (Davenport et al., 2016). In a retrospective study of heart transplantations, elevated ET-1 has been established as an independent predictor of accelerated cardiac allograft rejection (Parikh et al., 2019).

Sunitinib, a tyrosine kinase inhibitor, is already used for gastrointestinal stromal tumor and metastatic renal cell carcinoma, blocking PDGF and VEGF receptors (Chow and Eckhardt, 2007). In a rat kidney rejection model, orally administered Sunitinib was successfully used to prevent neointima hyperplasia, one hallmark of renal transplant arteriosclerosis (Rintala et al., 2016).

### Transmigration of Leukocytes Is a Multistep Progress

For immune cells, up-regulation of transmigration molecules on the surface of ECs is essential for migration from the circulation across the endothelial monolayer into the vessel wall. Before transmigration, leukocytes tether and roll along the EC monolayer, which is mediated by selectins and integrins (Muller, 2003). Subsequent leukocyte transmigration is mediated by specialized molecules (Muller et al., 1993). PECAM, CD99, or JAM-A are partially stored in lateral border recycling compartments (LBRCs) within ECs beneath the plasma membrane near endothelial junctions. To achieve sufficient transmigration, leukocytes are surrounded by LBRC membrane to provide unligated receptors for the immune cells (Mamdouh et al., 2003, 2009). Recent studies suggest relevance of IQ-domain GTPase-activating protein 1 (IQGAP1), bearing an actin-binding as well as a calmodulin-binding domain, for leukocyte transmigration. It has been shown that IQGAP1 interacts with LBRC, and knockdown of the protein prevents LBRC movement and leukocyte transmigration (Dalal et al., 2018, 2019). For the transient receptor potential canonical 6 (TRPC6), a ubiquitously expressed Ca^2+^ channel, co-localization with PECAM at endothelial junctions during transmigration has been proven. Chelation of Ca^2+^ as well as disruption of TRPC6 function stops leukocytes on the apical surface of ECs, suggesting a pivotal role for Ca^2+^ influx during transmigration. A TRPC6 function is likely located downstream of PECAM, because transmigration occurs after selective activation of TRPC6 and simultaneous PECAM blockade (Weber et al., 2015). Taking these findings together, interfering with Ca2+ currents might be a therapeutic approach for TV.

Leukocytes can either transmigrate paracellularly across ECs–ECs junctions or migrate transcellularly through single ECs (Vestweber, 2015). Paracellular transmigration requires loosening of endothelial junctions, with VE-cadherin as an important regulator of these junctional connections (Gotsch et al., 1997). To leave the bloodstream and invade the vessel wall, leukocytes must penetrate the basement membrane, which is composed of laminins and connects the endothelial monolayer with the underlying SMCs. Laminin 411 is ubiquitously expressed, whereas laminin 511 is expressed in distinct spots, and these spots are not preferred sites of leukocyte transmigration (Sixt et al., 2001). Laminin 511 induces VE-cadherin localization at endothelial junctions, which results in a RhoA-dependent stabilization of these cell junctions and reduced leukocyte transmigration (Song et al., 2017). Yeh and colleagues have shown that leukocytes are capable of generating 3D traction stresses to mechanically widen gaps between ECs and initiate transmigration (Yeh et al., 2018). Immunohistochemical analysis of the vessel wall of explanted organs with TV revealed that the majority of infiltrating cells are T cells. Macrophages account for 8–15% of infiltrating cells, whereas B cells and NK cells are encountered infrequently (van Loosdregt et al., 2006; Hidalgo et al., 2010).

**FIGURE 3:**
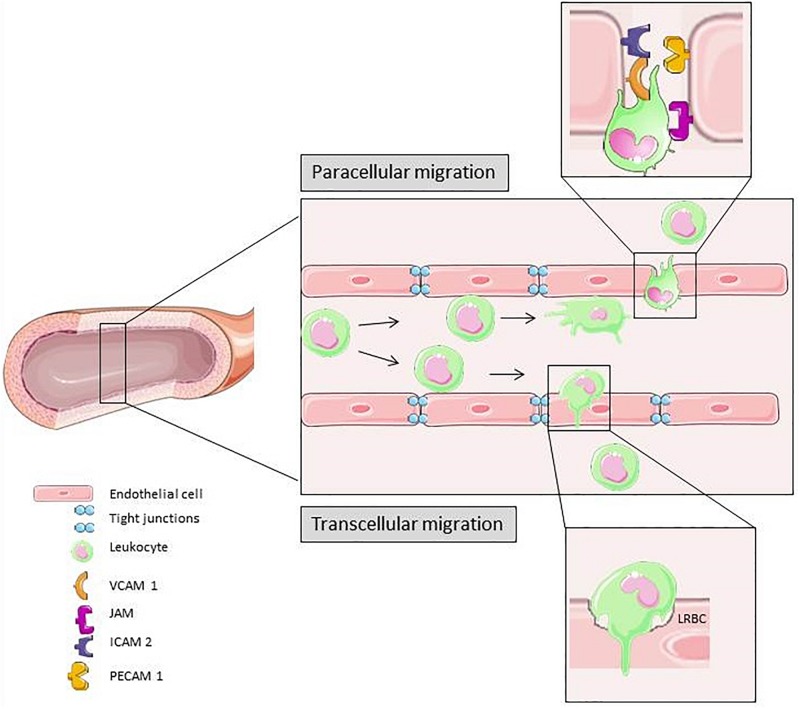
In response to different pro-inflammatory signals the leukocyte adhesion and transmigration cascade is activated. Slow down, rolling, firm adhesion and transmigration is mediated by different cytokines and molecules on the surface of leukocytes and ECs. Para-cellular transmigration through EC junctions is the primary route for extravasation (Vestweber, 2015). Elements of Figures 2, 3 and 4 were taken and adjusted from Servier Medical Art at http://smart.servier.com, licensed under a Creative Commons Attribution 3.0 Unported License.

Figure 3 is a schematic summary of transcellular and paracellular transmigration. The most prominent receptors involved in these two distinct pathways of leukocyte migration across the endothelial monolayer are depicted.

Prevention of leukocyte recruitment is a putative therapeutic intervention to prevent TV. It has been shown, that adding the heparin-based macromolecule Corline Heparin Conjugate (CHC) to the preservation solution forms a protective coating on the renal endothelium during cold storage after kidney explantation. Kidneys were analyzed 24 hours after transplantation, and the number of infiltrated leukocytes and the thrombotic area was significantly greater in control kidneys. CHC treatment is a promising strategy for prevention of I/R injury-induced leukocyte transmigration (Nordling et al., 2018).

A further means of delaying graft failure is to actively recruit suppressive, i.e., beneficial leukocytes into the graft. Application of depletional antibodies or the use of specific knockout and transgenic mouse strains enabled demonstration of the impact of different immune cell subtypes on ongoing graft failure. In various animal experiments, tolerogenic characteristics have been revealed for regulatory T cells (Tregs), T cells, B cells, NK cells, and NKT cells (Sakaguchi et al., 1995; Niimi et al., 1998; Seino et al., 2001; Yu et al., 2006; Haudebourg et al., 2007).

## Changes in Vascular Structure

Transplant vasculopathy is characterized by accumulation of extracellular matrix (ECM) (Lin et al., 1996), endothelial dysfunction, and VSMC proliferation, which result in diffuse, concentric intimal thickening (Rahmani et al., 2006). TV differs in appearance depending on the vessel structure: large coronary segments are affected by artery shrinkage, resulting in a loss of luminal diameter, whereas new intimal growth and subsequent loss of luminal diameter occurs in both large and small segments (Wong et al., 2001; Suzuki et al., 2010). In addition to the lumen narrowing, production of vasoconstrictors such as ET-1 and thromboxane is impaired and vascular resistance increases, which may result in ischemia (Rahmani et al., 2006). After activation, ECs elicit the differentiation and proliferation of quiescent medial VSMCs. VSMCs transdifferentiate from a contractile phenotype to dedifferentiated synthetic cells. Dedifferentiated VSMCs migrate from the media into the neointima and interstitial space, where they proceed to proliferate. VSMC proliferation and ECM production aggravate lumen narrowing. Furthermore, VSMCs produce cytokines which act in an autocrine fashion and strengthen proliferation (Michael, 2003; Dewald et al., 2005; Kennard et al., 2008; Wynn, 2008). Most neointimal muscle cells that evolved from VSMCs are similar in appearance to their medial progenitors; nevertheless, on the basis of some important functional differences, neointimal muscle cells are generally labeled smooth muscle-like cells (SMLCs) (Wong et al., 2001; Suzuki et al., 2010).

Not only ECs but also VSMCs can be directly affected by donor-specific anti-HLA I antibodies. *In vitro* stimulation with anti-HLA I antibodies induces VSMC proliferation in a dose-dependent manner. Also, migration is promoted, even in the presence of the proliferation inhibitor mitomycin C. As underlying mechanism for the observed effect, increased phosphorylation of FAK Tyr576, Akt Ser473, and ERK1/2 Thr202/Tyr204 is postulated (Li et al., 2011). In a humanized mouse model, human arteries were grafted into SCID/beige mice lacking functional T and B cell compartments. In this mouse model, passively transferred anti-HLA I antibodies were able to evoke neointima thickening and VSMC proliferation (Galvani et al., 2009). Recent studies demonstrated a pivotal role for sphingosine-1-phosphate (S1P) in anti-HLA I-induced intimal hyperplasia because treatment with anti-S1P antibodies and siRNA knockdown of sphingosine kinase-1 (SK1) inhibitor prevents intimal hyperplasia in mice (Trayssac et al., 2015). S1P is a mediator within signaling pathways for cell survival, proliferation, and migration (Herzog et al., 2010) and provides another potential therapeutic target for preventing TV (Spiegel and Milstien, 2003).

**FIGURE 4:**
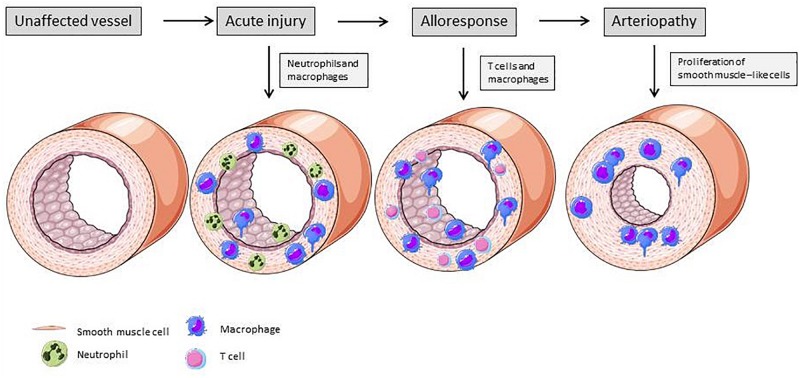
Changes of vascular structure due alloresponse following solid organ transplantation. Acute injury is mainly mediated by neutrophiles ans macrophages. These immune cells produce cytokines and induce expression of adhesion molecules on ECs, leading to further recruitment of immune cells. Activated ECs exert a pro-inflammatory phenotype and activate smooth muscle-like cells, resulting in their proliferation and lumen narrowing (Mitchell, 2009). Elements of Figures 2, 3 and 4 were taken and adjusted from Servier Medical Art at http://smart.servier.com, licensed under a Creative Commons Attribution 3.0 Unported License.

Figure 4 illustrates the vascular changes during alloresponse after solid organ transplantation. EC activation due to I/R injury predominantly leads to neutrophil recruitment into the vessel wall. At a later stage, lymphocytes and macrophages transmigrate into the vasculature and drive rejection. In the end, due to SMLC proliferation, TV with hallmark lumen narrowing is established.

## Effects of the Complement System on ECs

The complement system is part of the innate immune system and provides a link to adaptive immunity. It can be activated in three different ways: complement proteins bind to (1) antibodies bound to ECs, (2) proteins on cell membranes, or (3) carbohydrate residues on the surface. Independent of the mode of activation, all three pathways have in common protein C3 cleavage and subsequent membrane attack complex (MAC) formation. The MAC consists of complement proteins C5b-C9, and its activity results in cell lysis. Deposition of the complement fragment C4d on ECs was established as an independent marker for acute allograft rejection and as a predictor for long-term graft loss (Collins et al., 1999; Herzenberg et al., 2002; Racusen et al., 2003).

Nevertheless, there are some mechanisms that protect against EC damage due to an activated complement system. Expressed on human ECs, CD59 binds tightly into the forming MAC, thus preventing further MAC assembly (Davies and Lachmann, 1993). Another complement regulatory protein is CD55, which is also expressed on ECs. Incubation of ECs with CD55- and CD59-blocking antibodies induces complement fixation, which results in vWF release and platelet adhesion. Interestingly, complement fixation was increased in ECs from patients with type 3 von Willebrand disease lacking functional vWF. vWF seems to act as a complement regulator on the surface of ECs (Noone et al., 2016). Renal transplantation models in rats showed a decrease in mRNA expression of the complement regulators CD59 and Crry in allografts, and administration of anti-Crry and anti-CD59 antibodies results in reduced graft survival. A subsequent clinical study showed significantly increased graft survival in patients with high expression of complement regulatory proteins (Yamanaka et al., 2016).

It has been shown that assembly of MAC at sublytic levels has various effects in different cell types. *In vitro* stimulation of human ECs with C5b-C9 induces proliferation and migration. Furthermore, C5b-C9 promotes the release of pro-inflammatory cytokines, such as IL-6, MCP-1, and epidermal growth factor (Fosbrink et al., 2006), which contribute to recruitment of immune cells and ongoing inflammation.

In a mouse model of vascularized composite allografts, it was documented that the neutrophil and macrophage infiltrate was impaired in C3-deficient mice. Treatment with the C3 inhibitor CR2-Crry was associated with significantly prolonged graft survival (Zhu et al., 2017). Accordingly, the results of a clinical study in patients after kidney transplantation demonstrated that a local upregulation of C3 expression in glomeruli and tubuli was associated with ongoing acute cellular rejection (Serinsoz et al., 2005). Neutrophil recruitment to sites of inflammation can also be reduced by interfering with C5a and its receptor (Mueller et al., 2013).

## What Kind of Research Models Do We Have?

### Humanized Mouse Models

As there are several examples of successful therapy approaches in mice that failed to provide similar efficacy in humans, the transferability of preclinical animal studies to humans might seem doubtful (Mestas and Hughes, 2004). Species-specific differences between the murine and human immune systems should be taken into account. To overcome this limitation in the field of solid organ transplantation, the use of humanized mice to study human allografts and xenograft rejection may provide insights into the immune mechanisms responsible for graft rejection [recently reviewed by Kenney et al. (2016)].

Humanized mice have become an important preclinical tool in translational biomedical research (Walsh et al., 2017) and serve as a preclinical bridge in several fields [reviewed in Allen et al. (2019)]. Generally, these mice are reconstituted with human CD34^+^ stem cells derived from human cord blood, bone marrow, and peripheral blood (Lee et al., 2019). This is made possible by a targeted mutation in the interleukin 2 (IL-2) receptor common gamma chain [IL2rg(null)] in mice that are already deficient in T and B cells (Brehm et al., 2013). The most widely used immunodeficient strains engrafted with human hematopoietic cells are listed in Table 1 [modified after (Kenney et al., 2016); a more detailed list with immunodeficient mice that have been engrafted with human immune systems has been published elsewhere (Shultz et al., 2012; Hogenes et al., 2014)]. Among other immune defects, these animals do not develop functional NK cells. This allows efficient engraftment with human hematopoietic cells, generating a functional human immune system (Brehm et al., 2013).

**TABLE 1:** Immunodeficient mouse strains engrafted with human hematopoietic cells (modified after Kenney et al., 2016)

Strain	Abbreviation	*Il2rg* mutation	Characteristics	Immunological Characteristics	Availability [Refences]
NOD.Cg-*Prkdc*^*scid*^ *Il2rg^*tm1Wjl*^/SzJ*	NSG	Do not express the DNA repair complex protein Prkdc nor the X-linked Il2rg gene, the IL2rg^*null*^ mutation prevents cytokine signaling through multiple receptors	NOD strain. Immunodeficient and relatively radiosensitive due to a defect in DNA repair	Deficient in mature lymphocytes, serum Ig is not detectable and natural killer cell cytotoxic activity is extremely low	The Jackson Laboratory Stock: 005557 (Shultz et al., 2005)
NOD.*cg-Prkdc^*scid*^ Il2rg^*tm1Sug*^/JicTac*	NOG	Lacks the intracytoplasmic domain and will bind cytokines but will not signal	NOD strain. Immunodeficient and relatively radiosensitive due to a defect in DNA repair	Lacks T, B and NK cells, additional defects in innate immune cells	Taconic Bioscience Stock: CIEA NOG mouse (Ito et al., 2002)
NOD.Cg-*Rag1*^*tm1Mom*^ *IL2rg^*tm1Wjl*^/SzJ*	NRG	Rag1null mutation renders the mice B and T cell deficient and the IL2rg^*null*^ mutation prevents cytokine signaling through multiple receptors,	NOD strain. Extremely immunodeficient and relatively radioresistant	Lacks T, B and NK cells, additional defects in innate immune cells	The Jackson Laboratory Stock: 007799 (Pearson et al., 2008)
C.Cg-*Rag2*^*tm1Fwa*^ *Il2rg^*tm1Sug*^JicTac*	BRG	Lacks the intracytoplasmic domain and will bind cytokines but will not signal	Mixed background, predominately BALB/c strain: Immunodeficient and relatively radioresistant	Lacks T, B and NK cells, remaining innate immune cells are functional	Taconic Bioscience Stock: 11503 (Traggiai et al., 2004)

BLT(**b**one marrow, **l**iver, **t**hymus) humanized mice are generated by implantation of human fetal thymus and liver tissue into immunodeficient mice followed by systemic reconstitution with human innate (monocytes/macrophages, DCs, NK cells) and adaptive immune cells (B cells and T cells) (Wahl et al., 2019). The presence of a human thymic tissue allows human T cell education depending on HLA and the induction of HLA-restricted T cell responses in these mice is comparable with the human system (Wahl et al., 2019). Even though mice have a significantly shorter life span than humans, age-associated DNA methylation changes in the transplanted hematopoietic stem cells were not found to be increased (Frobel et al., 2018).

Recently, a humanized lung mouse model has been generated by subcutaneously implanting human lung tissue into the back of immunodeficient mice (Wahl et al., 2019). The human lung tissue vascularizes, expands and persists as a human lung implant. The engraftment of human non-hematopoietic cells, which are able to present antigens to autologous human immune cells in the full context of HLA (Wahl et al., 2019), will broaden the use of humanized mice for research in the field of transplantation.

### Heterotopic Versus Orthotopic Transplantation of Different Organs

Solid organ transplantation is an established treatment option for patients with end-organ dysfunction (Black et al., 2018). Progress in surgical techniques has minimized complications and reduced ischemic injury events. The more common orthotopic transplantation includes removal of the recipient’s organ and the insertion of the donor organ in the normal anatomic position, while in the case of heterotopic or “piggy-back” transplantation the diseased organ is retained.

Heterotopic heart transplantation (HHT) is extensively used in murine animal models in the non-working mode (Flecher et al., 2013). HHT in human patients, first performed by Barnard and Losman in 1974 (Barnard and Losman, 1975) is used rarely in comparison with orthotopic heart transplantation (OHT). The reason for this is major progress in immunosuppression therapy with the expansion of immunosuppressive protocols to dampen the host immune response and improve short- and long-term graft survival (Black et al., 2018). However, HHT may experience a renaissance, especially for children with advanced cardiomyopathy, where cardiac transplantation is limited by pediatric donor availability, by increasing the size of the donor pool. Beyond that, HHT also enables transplantations in adults previously not eligible for transplantation. It may be used especially in recipients with significant pulmonary hypertension (Flecher et al., 2013). Another advantage is that during temporary graft dysfunction due to early graft rejection the recipient heart could serve as an auxiliary pump (Holinski et al., 2016).

The prognosis for long-term graft survival and retention depends mainly on revascularization. Injury to the donor-derived microvasculature during organ explantation and subsequent ischemia may account for the documented clinical variability (Soares et al., 2015). Thereby, replacement of the donor graft vasculature by recipient-derived endothelial and endothelial progenitor cells may be a strategy for all non-vascularized free grafts or vascularization of tissue constructs engineered *in vitro* (Capla et al., 2006). Exogenous liposomal delivery of the angiogenic inducer VEGF gene prior to bone marrow–derived endothelial precursor cell transplantation has been shown to improve orthotopic liver transplantation-induced hepatic I/R injury (Cao et al., 2017). In this study, the transfer of the VEGF gene significantly increased hepatotrophic mitogen expression, in common with, for example, hepatocyte growth factor, angiogenesis, and NOS activity (Cao et al., 2017). In another study, the phosphodiesterase-5 inhibitor sildenafil citrate protected the graft microvasculature of warm ischemic kidney transplants (Lledo-Garcia et al., 2009) and autologous fat grafts (Soares et al., 2015). Sildenafil also decreased edema in lung I/R injury and ROS formation in a lung I/R injury model (Guerra-Mora et al., 2017).

The endothelial hypoxia-inducible factor HIF-2α has been shown to be essential for airway microvascular health and to play an important role in maintaining lung homeostasis (Jiang et al., 2019). In an orthotopic tracheal transplantation model, the genetic deletion of HIF-2α but not HIF-1α caused tracheal endothelial cell apoptosis. HIF-1α overexpression induced the expression of proangiogenic factors such as stromal cell-derived factor 1 (Sdf1) and VEGF, and promoted the recruitment of vasoreparative Tie2^+^ endothelial progenitor cells to the allograft (Jiang et al., 2019). These results are in line with the findings of a previous study using immortalized human microvascular endothelial cells (HMEC-1), demonstrating that reduction of both HIFs reduced cell survival, gene expression of glycolytic enzymes and pro-angiogenic factors compared with the corresponding control (Hahne et al., 2018).

## Accommodation: the Role of Protective Gene Expression in ECs

Accommodation in solid organ transplantation has been defined as stable allograft function without evidence of pathological alterations in the presence of alloantibodies and graft deposition of the complement component C4d (Smith and Colvin, 2012). The term accommodation was proposed at the beginning of the 1990s and was initially been demonstrated in the setting of xenotransplantation (Bach et al., 1991). It was later shown in a hamster-to-rat xenotransplantation model that increased expression of protective genes, i.e., anti-apoptotic and anti-oxidant genes, in ECs of the grafted organ was critical for mediating transplant survival (Bach et al., 1997). Moreover, it was found that regulation of endothelial gene expression patterns was accompanied by a host T_*H*_2 cell response. A follow-up study in a mouse-to-rat cardiac xenograft model demonstrated that the inducible anti-oxidant heme-degrading enzyme heme oxygenase-1 (HO-1) plays a key role in mediating anti-inflammatory protective effects. These protective effects were important for mediating transplant survival, possibly via the generation of the gaseous molecule carbon monoxide and biliverdin/bilirubin (Soares et al., 1998) [for a review see Soares et al. (1999)]. In accordance with these findings, Salama and colleagues showed, in studies on sensitized kidney transplantation patients with anti-HLA antibodies, that accommodation appears to be dependent on the expression of the anti-apoptotic gene Bcl-xL in the endothelium (Salama et al., 2001). Interestingly, this study demonstrates that low titers of anti-HLA antibodies can cause accommodation.

In a more recent HLA-mismatched, humanized murine HHT model, it was demonstrated that up-regulation of protective genes, including Bcl-2, Bcl-xL, and HO-1, was associated with protection against transplant rejection. Furthermore, expression of inducible inflammatory genes, e.g., ICAM-1 and VCAM-1, and pro-inflammatory cytokines such as IL-1β, TNF-α, and IL-6 was decreased in accommodated grafts (Fukami et al., 2012). In accordance with these findings, targeted up-regulation of HO-1 protected against anti-HLA class I antibody-mediated pro-inflammatory activation of ECs (Zilian et al., 2015).

Interestingly, a recent report compared the regulatory effects of interactions of the endothelium with antibodies against either AB0 or HLA antigens in a cell culture model of EA.hy926 ECs. It was demonstrated that ligation of ECs with anti-AB0 antibodies but not with anti-HLA antibodies caused accommodation (Iwasaki et al., 2012). The principal findings of these *in vitro* studies appear to be in accordance with a report on AB0-incompatible living kidney donor transplantation (Brocker et al., 2013).

Furthermore, ligation of anti-AB0 antibodies to EA.hy926 ECs induced upregulation of the complement regulatory proteins CD55 and CD59 on the RNA as well as on the protein level (Iwasaki et al., 2012). The first *in vivo* studies showed that overexpression of human CD55 and CD59 (hCD55, hCD59) protects mice from impaired kidney function in an experimental renal I/R injury model (Bongoni et al., 2017). A recent retrospective study of 150 patients after kidney transplantation, confirmed that lower intragraft expression of CD55 is a risk factor for rapid progression of chronic renal rejection (Cernoch et al., 2018). A more detailed study of kidney transplantations demonstrated a correlation between promotor polymorphisms in complement-regulatory proteins and graft survival (Michielsen et al., 2018).

## Conclusion

Graft rejection after transplantation of vascularized solid organs remains the main obstacle for graft survival. This complex disease pattern is caused by the interplay of different immune cell subsets and soluble factors from the recipient’s and the donor’s immune system. Graft rejection has heterogeneous characteristics, depending on the affected organ and whether it arises from cellular- or humoral-dependent pathways, but in any case, it leads to organ failure. While medication to treat acute rejection episodes is available, the therapeutic options for chronic rejection are limited.

The first target structure to be attacked after transplantation is the endothelium of the graft vessel wall. ECs are recognized by the recipient’s immune system due to the expression of surface molecules such as HLA and others, and activation of ECs is induced. Beside immunological components, brain death and I/R injury promote activation of donor-derived ECs. Activated ECs upregulate expression of various pro-inflammatory cytokines, and immune cells will be recruited. Various cytokines have been established as clinical markers to facilitate early diagnosis of graft failure and allow for treatment optimization. Furthermore, ECs present an amended expression pattern of adhesion and transmigration receptors on the surface to promote transmigration of leukocytes from the bloodstream across the EC monolayer into the vessel wall. Due to cytokines released from ECs as well as leukocytes, a pro-inflammatory microenvironment is built up and cannot be resolved.

The mononuclear infiltrate and growth factors further induce migration and proliferation of VSMCs and the resulting concentric intimal hyperplasia is a hallmark of long-term graft rejection. Crosslinking of antibodies on the surface induces phosphorylation and formation of intermediate signal transducers within the mTOR pathway, which regulates cytoskeletal changes, proliferation, and expression activity.

Further research is needed to gain deeper insight into how innate and adaptive immune responses contribute to graft rejection and how activation of ECs might be prevented. One therapeutic approach could be blocking ECs from presenting antigens, to prevent direct cellular cytotoxicity and to avoid synthesis of *de novo* donor-specific antibodies. Another option could be inhibiting recruitment of immune cells into the vessel wall, a concept of interest for other vascular diseases such as atherosclerosis. Humanized mouse models are important preclinical tools to study the underlying mechanisms of graft rejection. Despite many differences between the species, these mice represent a model system to evaluate new drugs and other treatment options without putting patients at risk.

## Author Contributions

LK and JL contributed conception and design of the Review. LK, MZ, VV, RA, SI, MW, and AW wrote separate sections of the manuscript. All authors conducted extensive literature research, read and approved the submitted version.

## Conflict of Interest

The authors declare that the research was conducted in the absence of any commercial or financial relationships that could be construed as a potential conflict of interest.
